# Incidence and Anatomical Properties of Retromolar Canal in an Iranian Population: A Cone-Beam Computed Tomography Study

**DOI:** 10.1155/2020/9178973

**Published:** 2020-03-09

**Authors:** N. Nikkerdar, A. Golshah, M. Norouzi, S. Falah-Kooshki

**Affiliations:** ^1^Department of Oral & Maxillofacial Radiology, Faculty of Dentistry, Kermanshah University of Medical sciences, Kermanshah, Postal Code: 6715847141, Iran; ^2^Department of Orthodontics, Faculty of Dentistry, Kermanshah University of Medical sciences, Kermanshah, Postal Code: 6715847141, Iran

## Abstract

**Objectives:**

Retromolar canal (RC) is an anatomic structure, and due to increasing demand for surgical procedure in the retromolar area of the mandible, the identification of the retromolar canal has become an issue of clinical concern. It can innervate the third molar and some of the muscles around the posterior segment of the mandible, complicating surgical procedures in the retromolar area and root canal treatment of third molars. The aim of this study was to evaluate the incidence and anatomical properties of RC in a western Iranian population using cone-beam computed tomography (CBCT) images. *Materials and Methods*. Two hundred bilateral CBCT images were collected and screened in the three spatial planes for the presence of an RC. Anatomical properties and location of the RCs were assessed according to their course and distance from the surrounding structures. The relationship between the presence of RC and age, sex, side, and presence of second and third molars was also evaluated. Independent samples *t*-test, ANOVA, Tukey's post hoc test, paired *t*-test, ANOVA, Tukey's post hoc test, paired

**Results:**

At least one RC was observed in 22% of the mandibles. Its bilateral incidence was 5.5%. Two major types of canals were detected, namely, type I, following a straight or curved course from the mandibular canal (MC) to the retromolar area (47.3%), and type II, coursing from the retromolar area to the radicular part of the third molar (52.7%). Regarding linear measurements, the mean RC diameter and the mean distance to the MC, second, and third molars were 0.68 ± 0.31, 13.7 ± 2.8, 15.3 ± 3.0, and 7.3 ± 2.3 mm, respectively.

**Conclusion:**

Based on the results of this study, RC was found in 22% of the cases; thus, it should be considered as a normal anatomical variation in the Iranian population rather than a rare finding.

## 1. Introduction

The mandibular canal (MC) and its branches mainly provide innervation and blood supply to the mandible and lower teeth. The mandible has multiple separate nerve canals in the embryonic stage of life, most of which, however, disappear or merge into one or more main canals in the next developmental stages [1]. The remaining canals can have different patterns varying from a single MC to a complex arrangement of multiple canals originating from the main MC or other structures.

It has been reported that there are many foramina with larger than 0.1 mm diameter in the surface of the posterior segment of the mandible. These foramina are sometimes connected to some canals or to a neurovascular plexus in the spongy portion of the mandibular bone and often have a connection to the inferior alveolar nerve or its dental branches. The largest foramina are found in the retromolar area [2], and their corresponding canals are referred to as the retromolar canals (RCs). These canals are sometimes considered as a type of bifid MC [3–11], but this assumption is not always true.

Several studies have reported different types of retromolar canals varying in their origin, course, and exit location [12–18].

Myelinated nerves and blood vessels comprise the contents of the RC according to microscopic studies [2, 17, 19, 20]. These nerves and vessels often originate from the inferior alveolar canal [12, 20, 21] and provide innervation and blood supply to the tendons of the temporalis and buccinator muscles, the most posterior parts of the alveolar process, and the second and third molar's gingival tissue [19, 20]. In some cases, the RC also contains the buccal and mylohyoid nerves [12, 22, 23].

Although the clinical significance of the RC has not been well studied, the fact that it may participate in the third molar innervation [20] can jeopardize successful endodontic treatment of this tooth. Presence of RC can also be considered as a major cause of failure in some mandibular nerve block injections, since some of the branches of the mandibular nerve enter the mandible through this canal [24]. Traumatization of the contents of this canal during tooth extraction, bone harvesting, and implant placement can also be problematic and cause bleeding, hematoma, and sensory impairment in the third molar area and buccal mucosa [22, 23, 25, 26]. In addition, tumors and infections of the retromolar area can spread to other areas through the retromolar foramina [27–29].

While there are many studies concerning the incidence of the retromolar canal, the methodologies and the study populations are different. In clinical practice, anatomical variations, such as supplemental or accessory canals and foramina, can only be detected by radiologic methods. However, conventional two-dimensional (2D) radiographs such as panoramic images are insufficient for detecting all anatomical structures, and in particular, the presence of an RC [14]. CBCT is now widely available, specifically for use in dentistry, and has been become notably effective for confirming anatomical variations of the mandibular canal that cannot be assessed on panoramic radiographs [3, 30].

Since there is no comprehensive study, which includes all types of RCs in the Iranian population, we conducted this study to evaluate the incidence and anatomical properties of the RC using cone-beam computed tomography (CBCT) images of a western Iranian population.

## 2. Materials and Methods

Bilateral CBCT images of 218 patients with the mean age of 46/42 ± 12/77 years were collected from a private oral and maxillofacial radiology clinic in Kermanshah, Iran. This study was approved by the Ethics Committee of Kermanshah University of Medical Sciences (Ir.kums.rec.1395.640). These images had been requested for third molar extraction, minor surgeries, implant placement, and orthodontic purposes. Eighteen images were excluded because of low quality (motion blur), large metal artifacts, large intraboney defects, and severe mandibular alveolar bone loss.

The images had been obtained by NewTom VGi (QR SRL Co., Verona, Italy) CBCT machine using 0.15 mm voxel size, 110 kVp, 10.88 mA, 5.4 s exposure time, and 12 × 8 inch field of view. Images were reconstructed in axial, coronal, and Panorex planes using the NNT viewer software 6.1.0 (NewTom©, QR S.r.l. Co., Verona, Italy).

**Figure 1:**
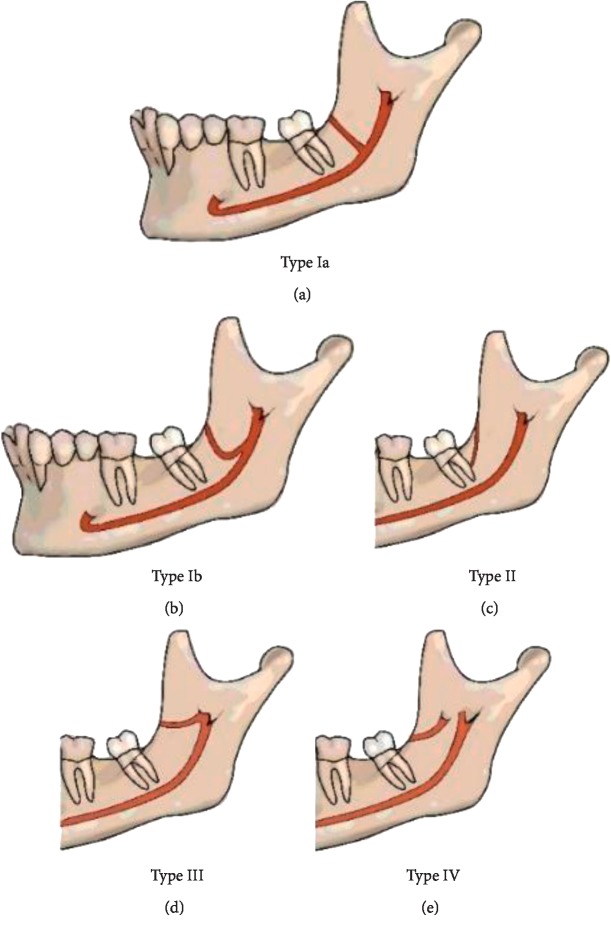
The four types of retromolar canals are as follows: *Type I*. The retromolar canal originates from the mandibular canal and courses toward the retromolar fossa through (a) a straight (Ia) or (b) a curved (Ib) trajectory. (c) *Type II*. The retromolar canal courses between the retromolar fossa toward the root portion of the third molar, with no connection with the mandibular canal. (d) *Type III*. The retromolar canal originates from the mandibular foramen and courses forward to the retromolar fossa. (e) *Type IV*. The retromolar canal originates from a foramen other than mandibular foramen and courses anteriorly toward the retromolar fossa.

**Figure 2:**
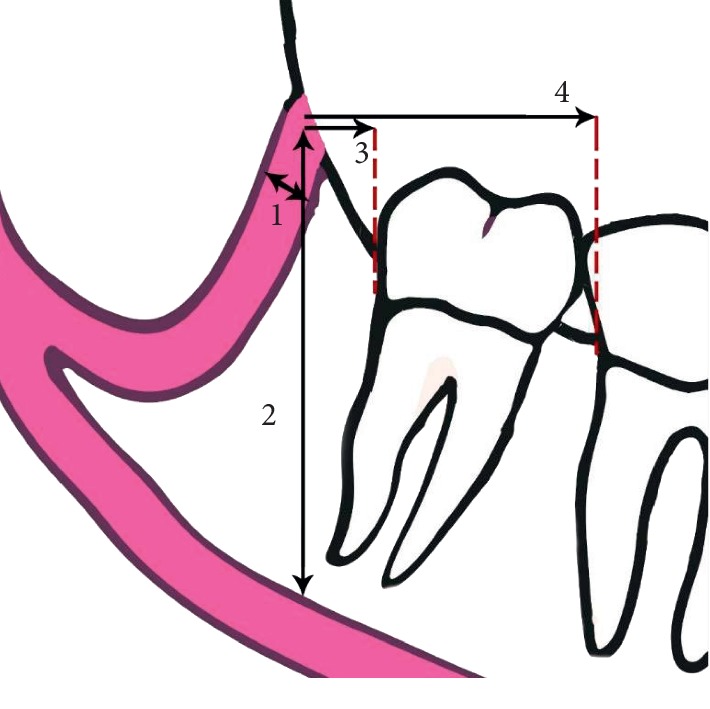
(1) The diameter of the RC at 3 mm below the center of its foramen. (2) The vertical distance between the retromolar foramen and the superior border of the MC. (3, 4) The distance between the mesial point of the retromolar foramen and the closest point of the third and second molars at the cementoenamel junction, respectively.

Based on previous studies, we classified the RC into four main types as shown in Figure 1 [12–18]. After recording the patient's sex and age, two calibrated oral and maxillofacial radiologists separately observed the Panorex reconstructions and recoded the presence of second and third molars. Then, the retromolar area was screened in various thicknesses in all spatial planes to find the RC.  Figure 2 shows the measurements made for the cases in which a RC was found.

Observations were made by two calibrated oral and maxillofacial radiologists. Each observer made the measurements independently. 2 weeks later, all measurements were repeated by the two observers. The interobserver agreement was calculated using Kappa statistics, and the Kappa coefficient was found to be 0.92. According to Cicchetti's classification, this value indicated excellent agreement between the observers [24]. The observations were made in a dimly lit room using a high-contrast 15.6-inch Full HD monitor (N56JR laptop PC, ASUS, Japan).

### 2.1. Data Analysis

For descriptive statistics, measures of central tendency and data distribution were calculated and analyzed. Normal distribution of the data for inferential analysis was evaluated by the Kolmogorov–Smirnov test. Independent samples *t*-test, ANOVA, Tukey's post hoc test, paired *t*-test, and chi-square were used to compare two groups, more than two groups, paired means, two dependent variables, and two qualitative variables, respectively. Pearson's correlation coefficient was used to evaluate the relationship between the qualitative variables.

The significance level was set at 0.05. SPSS 18.0 (SPSS Inc., Chicago, IL, USA) was used to analyze the data sets.

## 3. Results

Samples consisted of 95 males and 105 females with a mean age of 46.42 ± 12.77 years. The difference between the mean ages of the two sex groups was not significant (*p*=0.330).

Among 200 bilateral images, RC was observed in 44 cases (22%). It was more common in males than in females; however, this difference was not significant (*p*=0.289). The presence of retromolar canal did not show a statistical relationship with age (*p*=0.124).

Of a total of 400 sides studied, at least one RC was detected in 55 sides (13.8%), 28 in the right and 27 in the left side; 11 patients (5.5%) had bilateral RCs.

**Figure 3:**
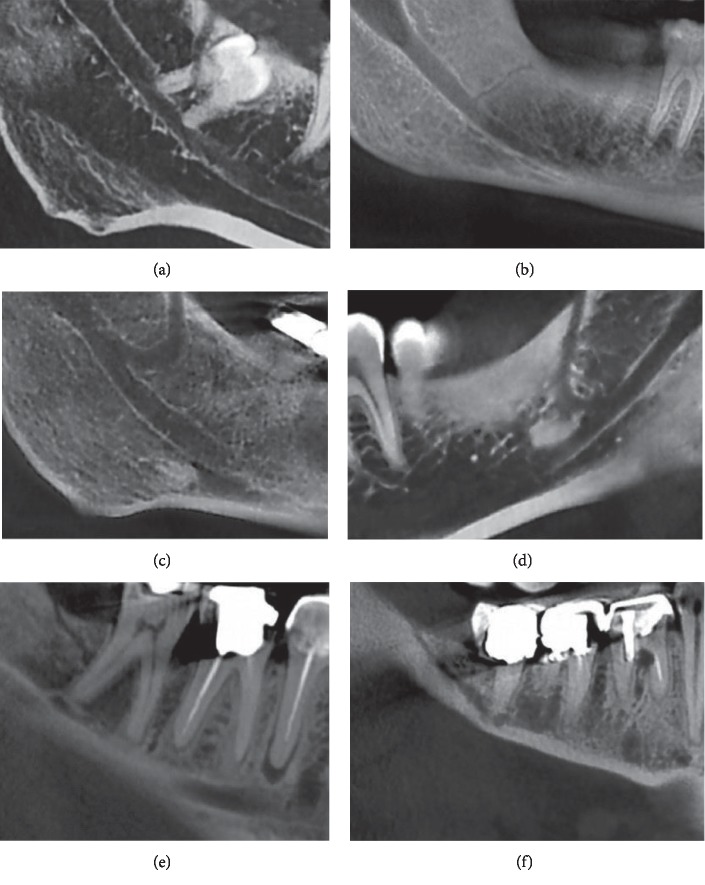
Some of the canals found in this study. (a), (b) Type Ia canals in two patients. Note the complicated relationship of the canal and third molar root in A. (c), (d) Two type Ib canals in two patients. (e), (f) Type II canals in two patients.

Figure 3 shows some of the canals found in this study. Type II was the most common type of RC followed by type Ib. No type III and type IV canals were found. Relative incidences of the retromolar canal types are reported in Table 1.

The mean diameter of the RC at 3 mm below the foramen was 0.68 ± 0.31 mm. This value was slightly higher in the left side than in the right side (*p*=0.610) and in males compared to females (*p*=0.121), but the differences did not reach statistical significance. The correlation between the canal diameter and age was not significant either (*p*=0.932 and *p*=0.012).

The mean diameter was significantly different between the canal types. Type II canals had the smallest mean diameter (0.53 ± 0.19 mm), while type Ib canals had the largest diameter (0.94 ± 0.38 mm).  Table 2 shows these results.

Presence of RC was not correlated with the presence of second molars. However, RC was more common in the sides where a third molar was present (*p*=0.007).

The location of the retromolar foramen was determined according to its distance from the mandibular canal and second and third molars; the results are represented in Table 3. None of these parameters were related to age (*p* > 0.05), and there was no significant difference between the two sides (*p* > 0.05) or males and females (*p* > 0.05), except for the mean distance of the retromolar foramen to the MC, which was significantly greater in males than females (*p*=0.01). Among the types found in this study, type Ib had the greatest mean distance from the third molar to MC and type II had the greatest mean distance from the second molar. These results are shown in detail in Table 4.

## 4. Discussion

This study evaluated the incidence and anatomical properties of the RC and foramen using CBCT images and presented a comprehensive classification of the RC types by reviewing the previous studies. RC can be defined as a canal that leads to one or more foramina in the retromolar area. The other end of the RC might be connected to the MC (type I), the root portion of the third molar (type II), the mandibular foramen (type III), or a separate foramen in the mandibular ramus (type IV).  Table 5 shows the variations in the population, methodology, and results of these studies [5, 13–19, 25, 28, 31–37]. The neurovascular content of the RC is an issue of clinical concern in surgical procedures involving the retromolar area [14]. Such an anatomic variation is clinically relevant for surgical procedures in the retromolar area such as removal of third molars, sagittal split osteotomy, bone harvesting in retromolar and ramus areas, and removal of cysts and tumors as well as for intraoral dental anesthesia [38].

Some studies considered the RC as a type of bifid MC, branching from the mandibular neurovascular bundle and coursing towards the retromolar fossa [39]. This definition is not always true since it does not include the type II canals found in this study and some previous studies [13, 15, 17, 18]. Considering this type is essential when comparing the results of studies because some authors did not include type II canals in their study.

As discussed earlier, a wide range of numbers has been reported for the incidence of the RC. Apart from the population differences, these inconsistencies can be due to different methodologies. In Japan, for example, there are three studies that reported three different numbers for the prevalence of RC (3.5, 53, and 75%) [12, 13, 35]. In a CBCT study, Jamalpour et al. reported a prevalence rate as high as 12.8% for the RC in an Iranian population [31]. Despite similar methodologies and populations, this number is lower than the results of the current study. This can be due to having different criteria, as they did not include type II RCs in their study and they only found canals corresponding to type I. If we do not include the type II, the prevalence of RCs reaches 12% in the current study, which is close to the results reported by Jamalpour et al. In another study on an Iranian population, Motamedi et al. reported that they found retromolar foramen in 40.4% of the studied cadavers, which is higher than the results of the current study [19]. A limitation for all anatomical studies that inspect the bone surface of the retromolar area is that they assess the foramina instead of the canals. It could be argued that some of the foramina located on the bone surface are not connected to any canals.

Patil et al. used high-resolution CBCT images (0.08 mm voxel size) and reported a 75.4% prevalence rate for RC [13], which is higher than the results of any other study. They also included type II canals in their assessment. High-resolution images can be very helpful for the detection of RCs but as reported by Patil et al., 75% of the canals found were type II canals, most of which were so narrow that their diameter could not be measured. The clinical importance of such fine canals is unknown and needs further research. Another reason for inconsistencies among the relevant studies is that some authors did not consider canals with diameters less than 0.5 mm [14, 16, 36] or even less than 1 mm [34] as RCs.

The results of the current study indicated that RC had an equal prevalence in males and females. This finding has been commonly reported in previous studies as well [12, 13, 33, 37, 40, 41]. In this study, bilateral retromolar canals were found in 5.5% of the cases, which have also been commonly reported by some previous studies [12, 15, 16, 25, 36, 41].

The most common type of retromolar canal in this study was type II, which is inconsistent with the results of Patil et al. [13]. However, Sisman et al. [15] reported that type I canals were more common than type II. Type Ib canals were found more frequently than type Ia in the current study. von Arx et al, on the other hand, reported that type Ia canals were more common than type Ib [25].

The mean width of the retromolar canal was 0.68 ± 0.31 mm at 3 mm below the center of the retromolar foramen. It should be noted that almost half of these canals were type II, most of which, had a diameter of 0.5 mm or less. von Arx et al. [25] and Han and Hwang [16] did not include type II canals and those smaller than 0.5 mm in width and reported the mean diameter to be 0.99 ± 0.31 and 1.13 ± 0.38 mm, respectively. Applying the same criteria to the subjects of the current study yielded a mean width of 0.98 ± 36 mm, which is very close to the results of the aforementioned two studies. Sisman et al. had the same inclusion criteria as in the current study, but they measured the width of the retromolar foramen and reported a greater diameter (1.64 ± 0.64 mm) [15] than ours; this could be due to the divergence of the canals as they approach the foramen.

Bilecenoglu and Tuncer did not find a correlation between the presence of the third molar in the dental arch and the incidence of the retromolar canal [36]. In our study, however, we found that retromolar canals tend to occur more commonly at sides where a third molar is present. This could be due to the high prevalence of type II canal, which has an anatomical connection to the radicular portion of the third molar. This canal may disappear after third molar extraction in the process of remodeling of the alveolar bone.

## 5. Conclusion

Based on the results of this study, RC was found in 22% of the cases; thus, it should be considered as a normal anatomical variation in the Iranian population rather than a rare finding. Since traumatization of this canal during the surgical procedures might result in excessive bleeding, hematoma, and sensory impairment, we suggest inspecting the retromolar area by CBCT prior to surgery.

## Figures and Tables

**Table 1:** Relative incidence of retromolar canal types.

		Frequency	Percent (%)
Type	Type Ia	10	18.2
	Type Ib	16	29.1
	Type II	29	52.7
	Type III	0	0
	Type IV	0	0
	Total	55	100

**Table 2:** Mean diameter of different retromolar canal types.

	Canal type			
	Type Ia	Type Ib	Type II	Total
Frequency	10	16	29	55
Mean	0.70	0.94	0.53	0.68
Std dev.	0.17	0.38	0.19	0.31
Min	0.40	0.40	0.30	0.30
Max	0.90	1.80	0.90	1.80

**Table 3:** Mean distance of retromolar canal to the mandibular canal and third and second molars.

	Mean	SD	Min	Max
Dist. to 3rd molar	7.3	2.3	2.6	11.4
Dist.to 2nd molar	15.3	3.0	7.7	21.4
Dist. to mand. canal	13.7	2.8	8.9	21.9

**Table 4:** Distance of different types of retromolar canal to third and second molars and mandibular canal.

	Canal type		Dist. to 3rd molar	Dist. to 2nd molar	Dist. to mand. canal
Type Ia	Frequency	Valid missing	4	4	10
			6	6	0
	Mean		5.425	12.225	11.780
	Std. dev.		2.7183	3.7393	1.5490
	Min		2.6	7.7	8.9
	Max		9.1	16.7	13.6

Type Ib	Frequency	Valid missing	2	6	15
			14	10	1
	Mean		10.500	14.450	14.467
	Std. dev.		1.2728	2.9126	3.2412
	Min		9.6	11.6	9.8
	Max		11.4	19.2	21.9

Type II	Frequency	Valid missing	17	18	27
			12	11	2
	Mean		7.376	16.300	13.956
	Std. dev.		1.8919	2.4217	2.6933
	Min		4.0	12.5	9.5
	Max		10.8	21.4	20.4

*p* value			0.028	0.029	0.048

**Table 5:** Prevalence of retromolar canal as reported in previous studies.

Author(s)	Year	Population	Number of subjects	Study method	% Prevalence
Jamalpour et al. [31]	2016	Iran	179	CBCT	12.8
Motamedi et al. [19]	2016	Iran	136	Anatomic	40.4
Park et al. [14]	2016	S. Korea	140	Anatomic	36.6
Capote et al. [32]	2015	Brazil	500	Panoramic	8.8
Sisman et al. [15]	2015	Turkey	947 hemi mandibles	CBCT	26.7
				Panoramic	0.03
Alves and Deana [18]	2015	Chile	86	Anatomic	18.6
Potu et al. [28]	2014	India	94	Anatomic	11.7
Muinelo-Lorenzo et al. [33]	2014	Spain	225	CBCT	36.8
				Panoramic	16.8
Han and Hwang [16]	2014	S. Korea	446	CBCT	8.5
Rashsurenet al. [5]	2014	S. Korea	755 hemi mandibles	CBCT	11.5
Patil et al. [13]	2013	Japan	171 (88 unilateral)	CBCT	75.4
Lizio et al. [34]	2013	Italy	233 unilateral images	CBCT	14.6
Rossi et al. [17]	2012	Brazil	222	Anatomic	26.5
von Arx et al. [25]	2011	Switzerland	121 (100 unilateral)	CBCT	25.6
				Panoramic	0.05
Kawai et al. [35]	2011	Japan	90 hemi mandibles of 46 cadavers	CBCT	52% of mandibles, 37% of sides
Bilecenoglu and Tuncer [36]	2006	Turkey	40	Anatomic	25
Narayana et al. [37]	2002	India	242	Anatomic	21.9

## Data Availability

We used data from patient's archives, so we considered patient privacy in this study.
